# Ecological determinants of effect of a free pit and fissure sealant program in Shanxi, China, 2017–2018

**DOI:** 10.1186/s12903-021-01821-8

**Published:** 2021-09-20

**Authors:** Xiangyu Wang, Wenjuan Ren, Yufang Li, Bin Zhao, Tingting Yang, Ruxia Hou, Junming Li, Junyu Liu

**Affiliations:** 1 Department of Pediatric and Preventive Dentistry, School and Hospital of Stomatology, Shanxi Medical University, Taiyuan, 030001 China; 2 Shanxi Province Key Laboratory of Oral Diseases Prevention and New Materials, Taiyuan, 030001 China; 3 School and Hospital of Stomatology, Shanxi Medical University, Taiyuan, 030001 China; 4 School of Statistics, Shanxi University of Finance and Economics, 696 Wucheng Road, Taiyuan, 030006 China

**Keywords:** Pit and fissure sealant, Oral disease prevention, Oral health care service, Spatial heterogeneity analysis, Medical resource

## Abstract

**Background:**

The aim of this study is to explore the spatial heterogeneity of the retention of PFS in children aged 7–9 years in Shanxi Province, North China and investigate the risk factors associated with PFS retention.

**Methods:**

In this study, 937 children aged 7–9 years from Shanxi Province, China were randomly selected, all of whom had at least one first permanent tooth sealed with PFS in 2016. The children were surveyed after 12 months (in 2017) and 24 months (in 2018). The Geo-detector model was used to explore the spatial heterogeneity of the retention rate of PFS and analyze the influence and interactions of the ecological factors on PFS.

**Results:**

3299 teeth from 937 children were analyzed. The PFS full retention rates after 12 months (in 2017) and 24 months (in 2018) were 81.6% and 75.1%, respectively. The incidence of caries of the first molar was 2.1% after 12 months and 5.4% after 24 months. The spatial heterogeneity of the PFS retention rate after 24 months was significant, which was shown as the retention rate of PFS increased from north to south after 24 months. Remarkably, the natural environmental factors exerted greater influence than the socioeconomic and medical resources factors after 12 months, where the interaction of fluorine in water (FW) had the strongest explanatory power of 52% (*P* < 0.05). The medical resources were important ecological factors after 24 months, and the percentage of medical technicians (PMT) had the strongest explanatory power of 70% (*P* < 0.05).

**Conclusion:**

The natural environmental factors and medical resources factors are important ecological factors determining the spatial pattern. The government should strengthen medical and technician construction in North China, comprehensively control fluoride in water, optimize the allocation of medical resources, and promote the balanced development of regional medicine.

## Background

Dental caries are one of the ten most prevalent chronic conditions [1]. Despite the widespread decline in the prevalence and severity of caries in permanent teeth in high-income countries over the past few decades [2, 3], the prevalence and burdens of caries are severe across the world [4]. In the United States, the 2013–2014 National Health Nutrition and Examination Survey indicated that 33.4% of adults aged 21–64 years had untreated coronal dental caries [5]. Moreover, according to China’ s Fourth National Oral Health Epidemiological Survey (2017), 70.1% of 5-year-olds had primary dental caries and 34.5% of 12-year-olds had experienced dental caries in their permanent teeth [6]. If left untreated, dental caries can result in pulpitis and pain, adversely affecting student quality of life. In addition, the treatment of oral diseases is costly, imposing a significant financial burden on both individuals and national health care systems [7, 8].

Pit-and-fissure sealants (PFS) are instrumental in managing pits and fissures of the occlusal surface. Further, there is evidence to suggest that sealants reduce the incidence of caries by 76% on sound occlusal surfaces (compared to the nonuse of sealants) during the 2–3 year follow-up period [9, 10]. Although PFS has been shown to be effective in preventing dental caries, few people in developing countries (such as China) are willing to cover the cost themselves and most health insurance companies refuse to cover oral health prevention in children.

The National Oral Disease Intervention Program for Children (NODIPC) introduced by the government in 2008 has provided free oral health prevention services to children in China [11]. In this program, some public hospitals and oral clinics (as project implementors) are obliged to provide primary school children with oral health prevention services such as oral check-ups, oral hygiene instructions, and most importantly, free PFS for some children aged 7–9 years. Since 2008, more than 1.21 million molars have been sealed by PFS in the Shanxi Province. The project covers all 11 cities in North China's Shanxi Province, the geographical stratification of which includes the northern region (Shuozhou City and Datong City), the central region (Luliang City, Yangquan City, Jinzhong City and Taiyuan City), the southeastern region (Jincheng City and Changzhi City), and the southern region (Yuncheng City and Linfen City).

It is somewhat surprising that although most research has proved the effectiveness of PFS in ideal environments, the rate of dental caries among Chinese children has increased since NODIPC was implemented in 2008 [10, 12, 13]. The effectiveness of PFS in real life might be lower than in ideal experimental conditions. This inspired us to explore the effectiveness and influencing factors of PFS. Further, most previous studies on factors affecting PFS only discussed the impact of a single ecological factor, lacking consideration of interactions between two or more ecological factors on PFS retention. Accordingly, the aim of our study was to explore the spatial heterogeneity of the retention of PFS after 12 and 24 months in children aged 7–9 years within the NODIPC project in Shanxi Province, China. Additionally, the risk factors associated with PFS retention were investigated.

## Methods

### Study design, setting and participants

A program-based cohort study design was employed to assess the effect of a free PFS and its ecological determinants from 2017 to 2018. In China, NODIPC is a livelihood project that provides prevention and treatment programs for oral diseases in children. It is subsidized by the government [11]. The government publicizes the program through schools. Children accompanied by their parents were volunteered to participate in the NODIPC. The government organize pediatric dental experts to monitor the quality of the program of NODIPC every year. In the study, all children age 7–9 years randomly selected from the project were study populations, and the dental doctors and nurses in the NODIPC were the sealant performer. The retention of PFS was assessed by the same examiner who is one of experts with great professional knowledge. Further, the protocol and consent forms for this study were reviewed and approved by the Ethics Committee of Shanxi Medical University.

### Sample size and sampling procedure

The sample size calculation was as follows. Firstly, the initial sample follows this formula: n_1_ = (t^2^PQ)/d^2^. Here, P is the caries rate of first permanent tooth, and d is the allowable error. *P* was 0.5 for lack of research on the caries incidence rate of first permanent molars in Shanxi Province. When d = 0.1P, the level of significance = 5%, the initial sample was 384 through calculation. Secondly, count effect adjustment: n2 = n1 × deff = 768. Thirdly, considering the factors such as lost follow-up and database information error, the sample size is increased by 20% in order to ensure it sufficient, and the minimum sample size was 922 through calculation. Ultimately, for the convenience of sampling samples from 11 cities in Shanxi Province, the actual sample size was 937.

The sample were selected using stratified random sampling method from participants. The details are as follows. In NODIPC, project executors must register participants' information such as name, telephone number, ID number and school. All information were accurately entered into the NODIPC’s management system by computer in a timely manner. In the survey, samples of each city were randomly selected from the system in equal proportion according in 2016 and been evaluated in 2017and 2018.

### Data collection and data quality assurance

**Fig. 1:**
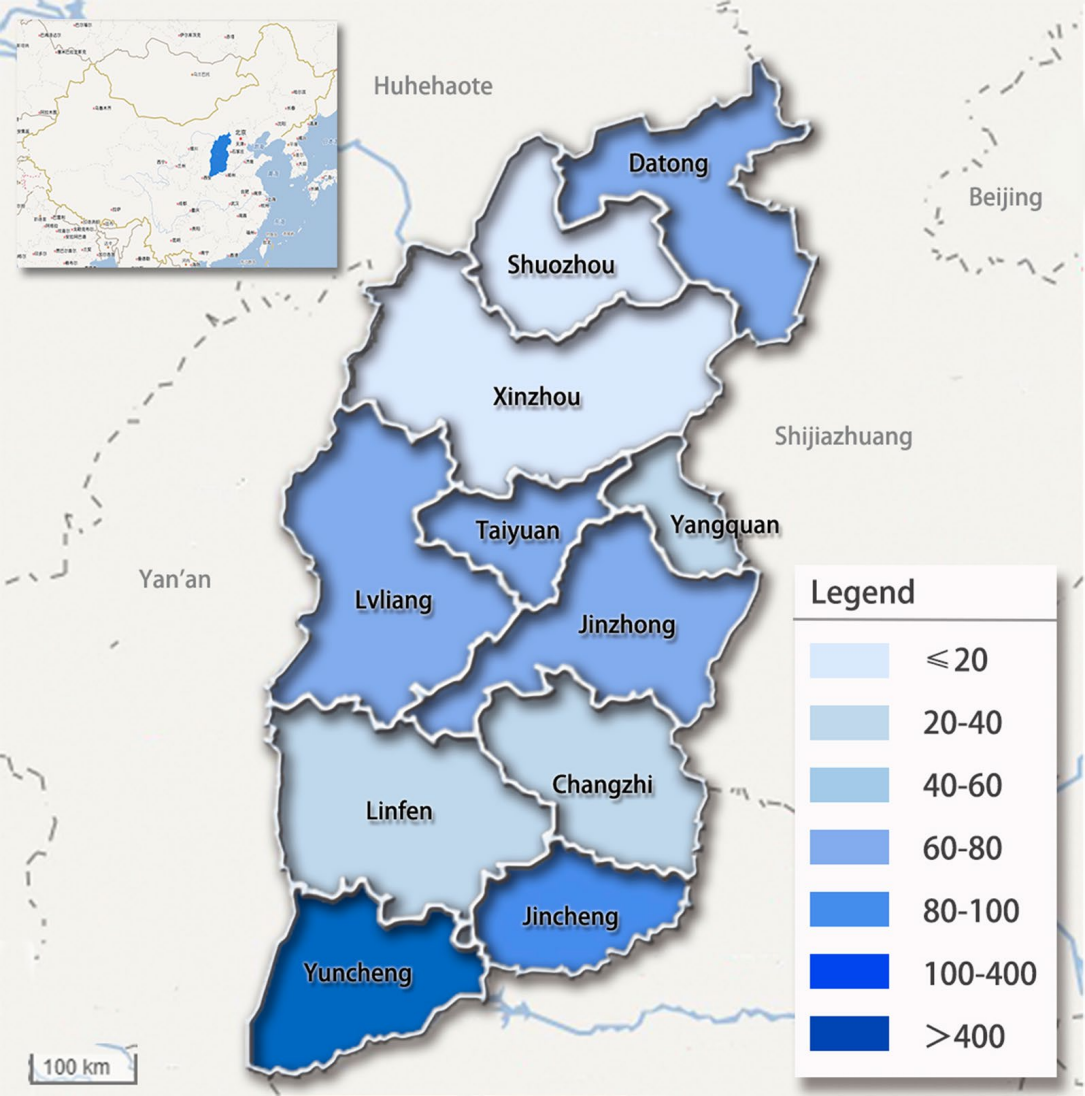
Number of children sampled in each city of Shanxi province (source of base map: https://map.baidu.com)

For this study, 937 children aged 7–9 years were randomly selected from the project who had at least one first permanent tooth sealed with PFS in 2016. The number of children sampled in each city is displayed in Fig. 1. Written informed consent for involvement in the study was obtained from all participating children and their parents. The PFS retention was reviewed after 12 months (in 2017) and 24 months (in 2018).

All dentists and assistants on the program are fully trained and qualified. The government organizes training sessions for sealant performer (dental doctors and nurses) in provincial cities annually. All sealant performer will gather at the sessions to learn the techniques and pass the examination. In addition, the government conducts mid-term and final project supervision annually to ensure that the performer's operation is standardized.

The selected children’s first permanent molars had been sealed with PFS in hospitals and dental clinics in a project run by NODIPC, during which routine clinical procedures for fissure sealant application were followed. The first permanent molars (PFS indicators) were cleaned with a dental rotary brush, etched with 37% phosphoric acid using cotton rolls for moisture control, and then sealed by a light-polymerized resin-based sealant.

Indications for PFS include the following: a permanent molar has completely erupted with a deep or irregular fissure and pit (especially if a probe tip can be inserted or becomes stuck); or the contralateral tooth (or a tooth with the same name) is carious or restored. Contraindications for sealant use include the following: a permanent molar has incompletely erupted; dental caries or filled teeth; teeth possessing a good self-cleaning effect with no deep fissures or pits on the surface; and children who were not able to cooperate during the procedure. The buccal/palatal surfaces of 1841 teeth of 937 children were not sealed because of incomplete exposure of buccal/palatal fissures and pits or for other reasons.

Examinations took place in 2017 and 2018 at 12 and 24 months after PFS. The children were examined in their schools using appropriate equipment (disposable gloves, mirrors, and probes). A headlamp (or flashlight) was used in conjunction with the natural classroom lights. Occlusal surfaces and the buccal/lingual surface of teeth were both considered. The retention of PFS was assessed by the same examiner, where sealant retention was clinically evaluated as full retention, partial retention, or total loss. The incidence of caries was recorded using the following: decayed, filled, or full coverage (such as a stainless-steel crown). Ultimately, 3299 sealed teeth of 937 children (including 3299 occlusal surfaces and 1458 buccal/palatal surfaces) were examined to study the PFS reservation and influencing factors.

### Ecological determinants of PFS

**Fig. 2:**
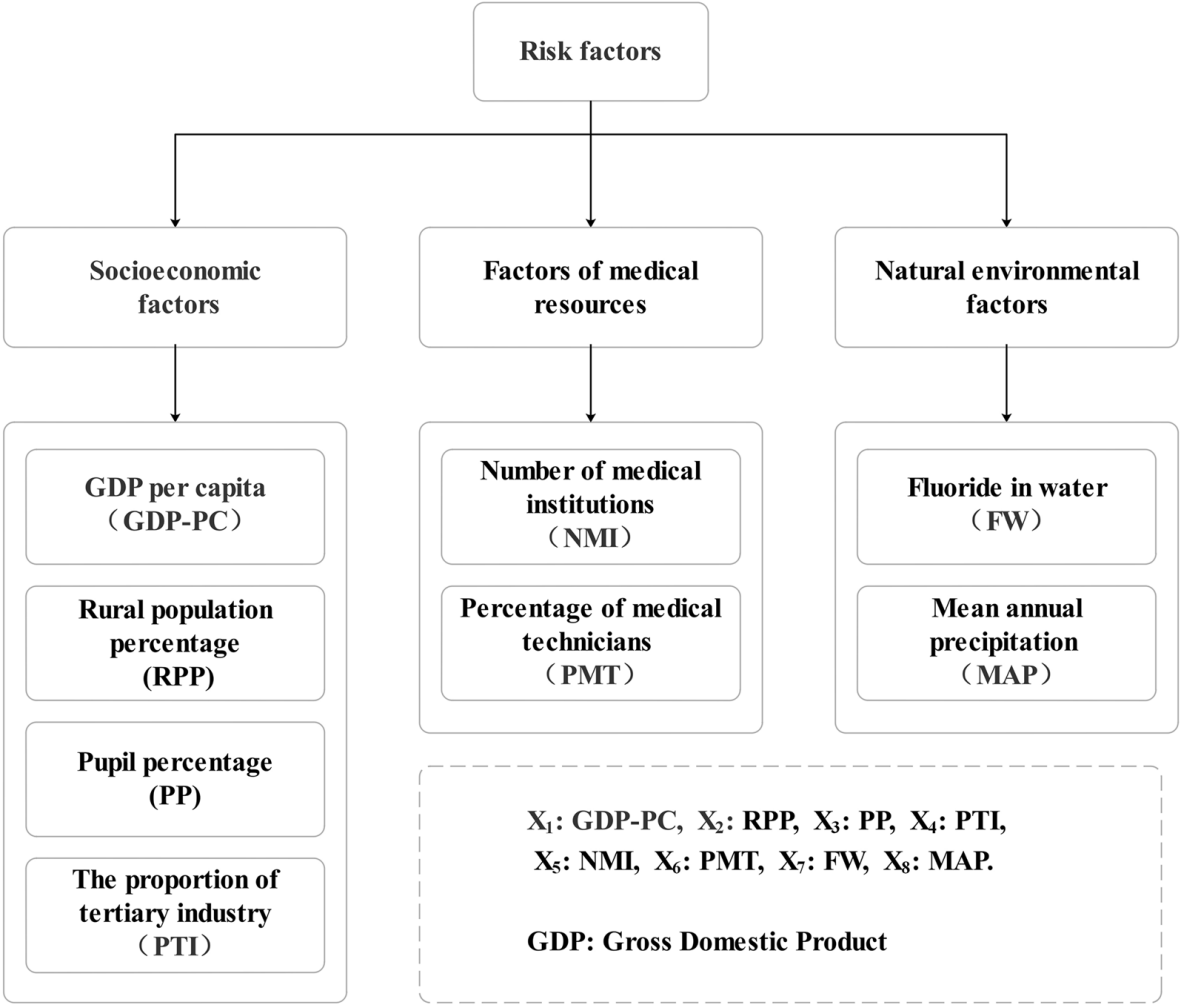
The three types of ecological risk factors and their proxy variables on PFS

Most previous studies [14–16] on the influencing factors of PFS only discussed the impact of a single factor, ignoring the interaction of two or more ecological factors on PFS retention. Accordingly, we explored the relationship between PFS retention rate and factors such as gender, region, and the dental face sealed by PFS and the interaction influence of socioeconomic, medical conditions, and natural environmental factors on PFS retention rate. We selected eight specific variables (Fig. 2) based on the availability of data. Socio-economic factors had four variables: GDP per capita (GDP-PC), rural population percentage (RPP), pupil percentage (PP), and tertiary industry percentage (PTI). There were two variables that influenced medical conditions: the number of medical institutions (NMI) and the percentage of medical technicians (PMT). Further, there were two variables for the natural environment: fluorine in water (FW) and mean annual precipitation (MAP). The first two ecological factors were obtained from the 2017 and 2018 Statistical Yearbooks of Shanxi Province, FW was obtained from the municipal water company, and MAP was obtained from a weather website (http://www.cma.gov.cn/).

### Statistical model

Each examined tooth surface was considered an independent sample during analysis, and their PFS retentions were recorded using double-entry to create a database. The frequency and percentage of each type of surface condition was calculated and the chi-square test was employed to analyze the dental position, gender, region, and dental face. The q-statistics test was also used to analyze the spatial heterogeneity of PFS retention rate and risk factors.

The Geo-Detector model was first presented by Wang [17] in 2010. Moreover, the q-value estimated from the Geo-Detector model is used in measure the degree of spatial stratified heterogeneity [18, 19]. The theory of the Geo-Detector model is that two variables would be (linearly or non-linearly) coupled in strata if one causes another or if they are associated. The Geo-Detector model differs from ordinary linear regression models in that the former will investigate non-linear associations and any interaction effects between various variables [20].

The magnitude of the q-value (q) can quantify the influencing power of a single factor (or interaction among the various factors) and can be calculated with the following formula:

$$\begin{aligned} {\text{q}} & = 1 - \frac{{\sum\nolimits_{h = 1}^{L} {\sum\nolimits_{i = 1}^{{N_{h} }} {\left( {Y_{hi} - \overline{Y}_{h} } \right)^{2} } } }}{{\sum\nolimits_{i = 1}^{N} {\left( {Y_{i} - \overline{Y}} \right)^{2} } }} \times 100\% \\ & = 1 - \frac{{\sum\nolimits_{h = 1}^{1} {N_{h} \sigma_{h}^{2} } }}{{N\sigma^{2} }} \times 100\% \\ \end{aligned}$$

Here, N is the number of spatial lattice pixels over the Shanxi province stratified into the *h* = 1, 2, …,* L* stratum according to influencing factors (*Xs*); stratum *h* includes *N*_*h*_ spatial statistical pixels; and *Y*_*i*_ and *Y*_*h*i_ denote the retention rate of PFS of the *i*th pixel and in stratum *h* of the influencing factors (*Xs*) separately. Terms *Y* and *σ*^2^ represent the common mean and variance of retention rate of PFS over the Shanxi province, respectively. It should be noted that the range of q-values is between 0 and 100% and the larger the q-value, the stronger the influence of variable *X* on *Y*.

The q-value was calculated based on the cross-classified stratum of two different factors: X_1_ and X_2_, q(X_1_ ∩ X_2_). This value can identify the interaction effects of X_1_ and X_2_ on the dependent variable (Y). The Geo-Detector model provides the judging rules to assess the types of the interaction effects of X_1_ and X_2_ have on Y (Table 1) [17, 20, 21]. The model can also identify whether the two factors (X_1_ and X_2_) weaken or enhance the influence on Y. The interaction effects can be explained as five types of interactive relationships, and the specific judging rules are listed in the Table 1.

**Table 1:** The interactive categories of two factors and the interactive relationship

Judging rules	Types of interaction effects
q(X_1_ ∩ X_2_) < Min(q(X_1_), q(X_2_))	Non-linearly weakened
Min(q(X_1_), q(X_2_)) < q(X_1_ ∩ X_2_) < Max(q(X_1_), q(X_2_))	Univariate non-linearly weakened
q(X_1_ ∩ X_2_) > Max(q(X_1_), q(X_2_))	Bivariate enhanced
q(X_1_ ∩ X_2_) = q(X_1_) + q(X_2_)	Independent
q(X_1_ ∩ X_2_) > q(X_1_) + q(X_2_)	Non-linearly enhanced

## Results

### Descriptive statistics

An analysis of 3299 teeth from 937 children was performed. The demographic characteristics of the participants and the retention rates of sealants are displayed in Table 2. The PFS full retention rates after 12 months (2016–2017) and 24 months (2017–2018) were 81.6% (n = 2693) and 75.1% (n = 2478), respectively. The partially retained and total loss rates of PFS were 13.4% (n = 443) and 4.9% (n = 163) after 12 months (2016–2017) and 14.2% (n = 468) and 10.7% (n = 353) after 24 months (2017–2018), respectively.

**Table 2:** Baseline characteristics of the participants

	Retention of PFS (%)			*P*
	Full retention	Partial retention	Total loss	
*12 months (in 2017)*	2693 (81.6)	443 (13.4)	163 (4.9)	
Area				
Urban	1788 (81.2)	305 (13.9)	108 (4.9	0.59
Rural	905 (82.4)	138 (12.6)	55 (5.0)	
Sex				
Boy	1464 (82.1)	240 (13.5)	80 (4.5)	0.42
Girl	1229 (81.1)	203 (13.4)	83 (5.5)	
Tooth arch				
Upper	1427 (83.0)	208 (12.1)	85 (4.9)	0.06
Lower	1266 (80.2)	235 (14.9)	78 (4.9)	
Tooth surface				
Occlusal surface	2905 (88.1)	223 (6.8)	171 (5.2)	0.00
Buccal/Palatal surface	1172 (80.4)	66 (4.5)	220 (15.1)	
*24 months (in 2018)*	2478 (75.1)	468 (14.2)	353 (10.7)	
Area				
Urban	1614 (73.3)	319 (14.5)	268 (12.2)	0.00
Rural	864 (78.7)	149 (13.6)	85 (7.7)	
Sex				
Boy	1345 (75.4)	268 (15.0)	171 (9.6)	0.04
Girl	1133 (74.8)	200 (13.2)	182 (12.0)	
Tooth arch				
Upper	1310 (76.2)	238 (13.8)	172 (10.0)	0.29
Lower	1168 (74.0)	230 (14.6)	181 (11.5)	
Tooth surface				
Occlusal surface	2692 (81.6)	260 (7.9)	347 10.5)	0.00
Buccal/Palatal surface	1064 (73.0)	85 (5.8)	309 (31.2)	

Table 2 indicates there was no statistically significant differences in area (*P* = 0.59), sex (*P* = 0.42), and tooth arch (*P* = 0.06) in 2017. In comparison, the difference in PFS retention rate between boys and girls in 2018 was statistically significant (*P* = 0.04); moreover, the retention rate of boys was higher. The difference in PFS retention rate between urban and rural areas was statistically significant (*P* = 0.00) in 2017, and the retention rate in rural areas was higher than that in urban areas. The retention rate of the occlusal surface was significantly higher than that of the buccal/palatal surface in 2017 and 2018 (*P* = 0.00 and *P* = 0.00, respectively). Further, no statistically significant differences were found in the retention rate between upper and lower first permanent molars in 2017 and 2018 (*P* = 0.06 and *P* = 0.29, respectively).

**Fig. 3:**
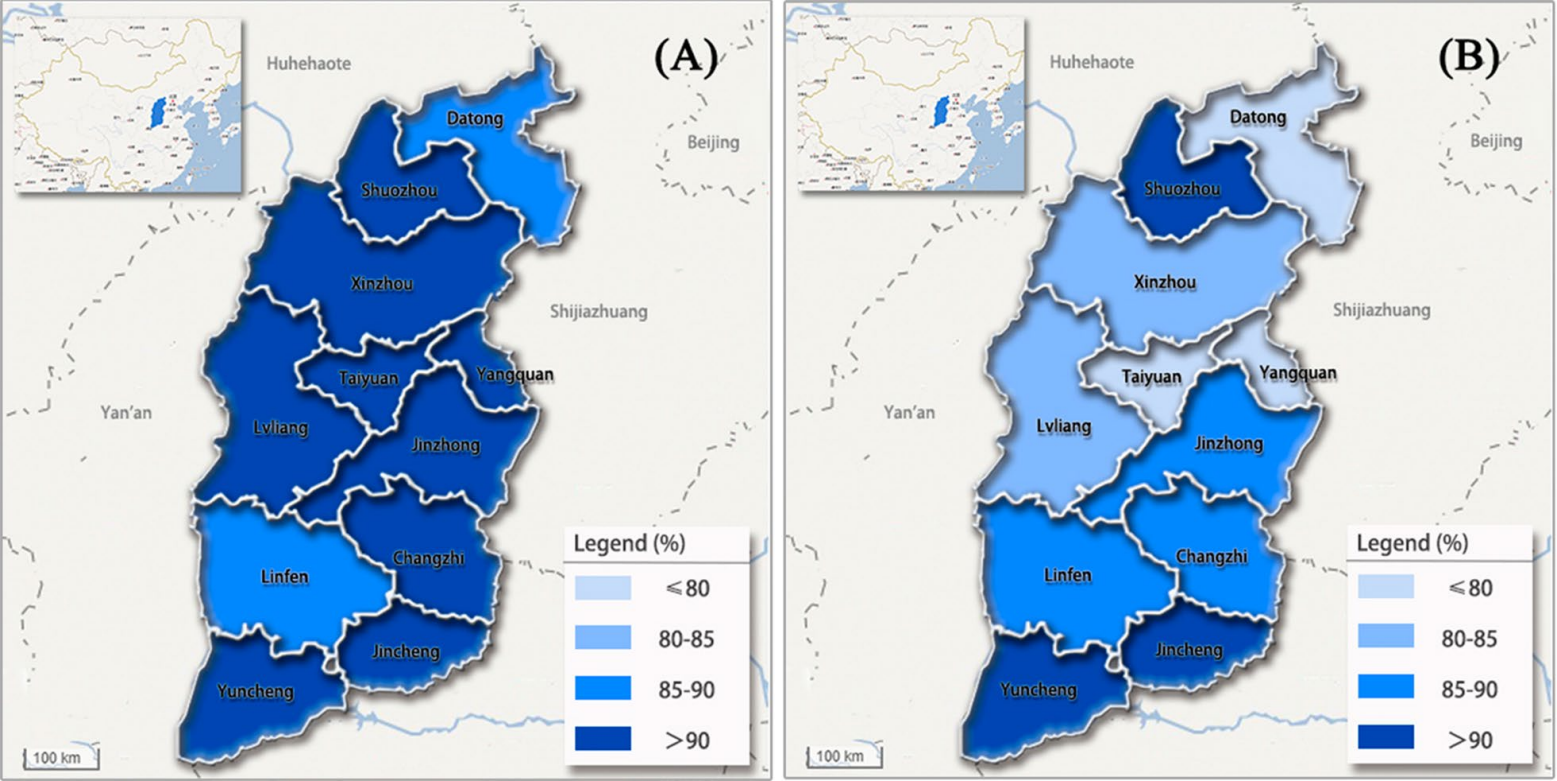
Spatial distribution of PFS retention after 12 months in 2017 (**a**) and 24 months in 2018 (**b**) ( source of the base map: https://map.baidu.com)

The Geo-Detector model was used to study the spatial heterogeneity of the retention rate of PFS in 11 cities of the Shanxi province in 2017 and 2018. The PFS retention rate was divided into four levels (< 0.80, 0.80–0.85, 0.85–0.90, and 0.90–1.00) to compare the difference across regions. The spatial distribution of the retention rate is shown in Fig. 3.

### Univariate analysis

**Fig. 4:**
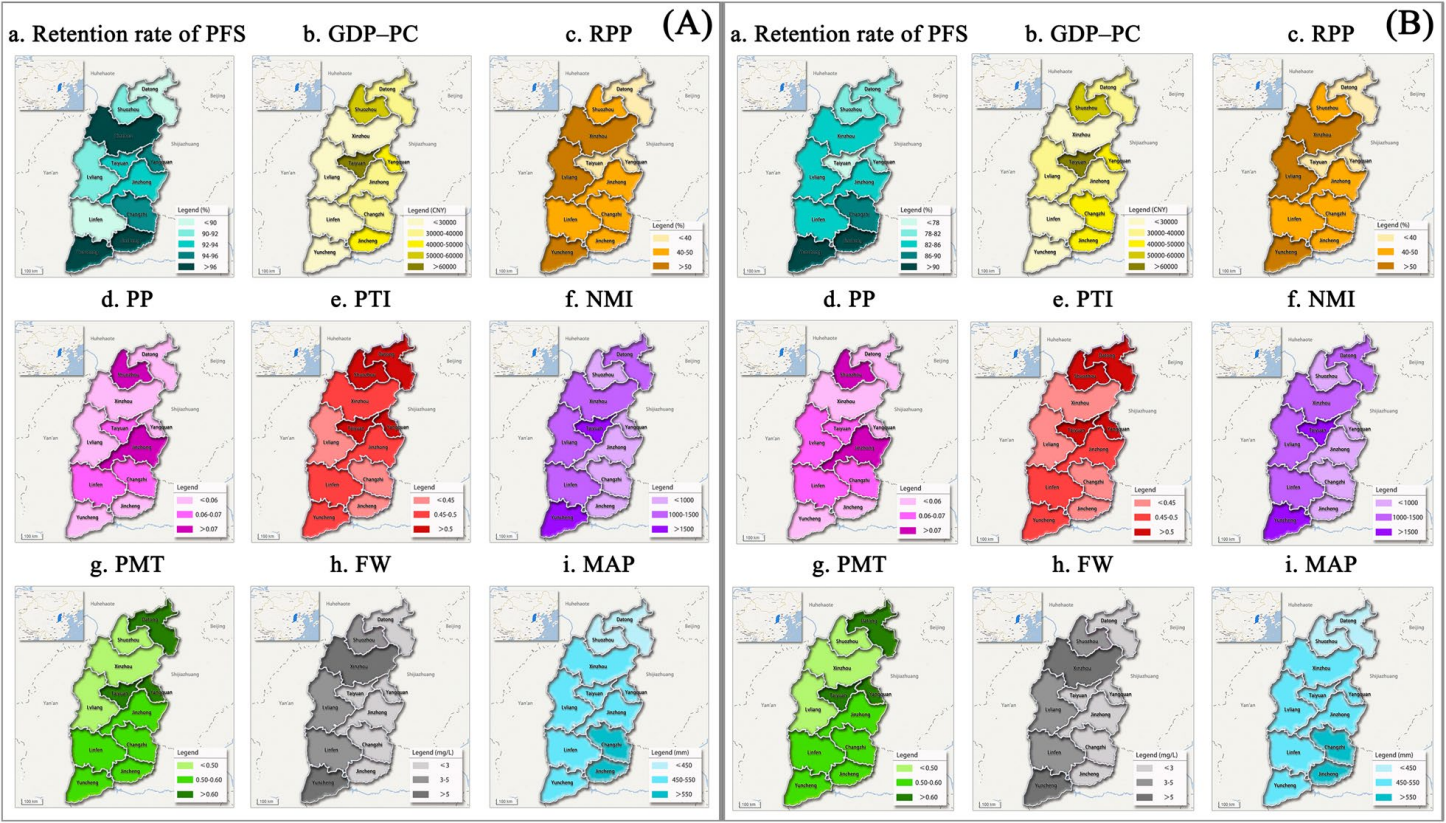
The spatial heterogeneity of ecological factors and PFS retention rate after 12 months in 2017 (**a**) and 24 months in 2018 (**b**) ( source of the base map: https://map.baidu.com)

Table 3 lists the Geo-Detector q-values of the three types of ecological factors (socioeconomic, medical resources, and natural environment) estimated by the Geo-Detector model. Table 4 shows the q-values of the eight specific ecological factors, while Fig. 4 shows the spatial heterogeneity of ecological factors and PFS retention rate. Generally, the results indicated that the natural environmental factors had a greater influence on the retention rate of PFS (q = 0.43, *P* < 0.05) compared to socioeconomic resources and medical factors in 2017. The q-value of FW was the highest (q = 0.52, *P* < 0.05) in 2017, indicating that FW had the strongest explanatory power of 52% for the spatial heterogeneity of PFS retention rate after 12 months, followed by MAP. After 24 months, the influence of medical resource factors on the retention rate of the PFS (q = 0.68, *P* < 0.05) was greater than the socioeconomic and natural environmental factors in 2018. Finally, the q-value of PMT was the largest (q = 0.70, *P* < 0.05) in 2018, indicating that PMT had the strongest explanatory power of 70% for the spatial heterogeneity of the PFS retention rate after 24 months, followed by the RPP.

**Table 3:** Q-values of the three ecological factors

Factors	Q	*P*
*12 months (in 2017)*		
Socioeconomic factors	0.24	0.03
Medical resources factors	0.22	0.04
Natural environmental factors	0.43	0.04
*24 months (in 2018)*		
Socioeconomic factors	0.45	0.03
Medical resources factors	0.68	0.03
Natural environmental factors	0.53	0.04

**Table 4:** Q-values of the eight specific ecological factors

Ecological factors	Q	*P*
*12 months (in 2017)*		
Socioeconomic factors		
X_1_	0.14	0.04
X_2_	0.07	0.04
X_3_	0.11	0.03
X_4_	0.12	0.04
Medical resources factors		
X_5_	0.13	0.04
X_6_	0.06	0.03
Natural environmental factors		
X_7_	0.52	0.03
X_8_	0.36	0.36
*24 months (in 2018)*		
Socioeconomic factors		
X_1_	0.11	0.04
X_2_	0.69	0.01
X_3_	0.35	0.04
X_4_	0.29	0.03
Medical resources factors		
X_5_	0.14	0.01
X_6_	0.70	0.03
Natural environmental factors		
X_7_	0.15	0.04
X_8_	0.10	0.04

### Multivariate analysis

In this study, the interaction influence of socioeconomic, medical resources, and natural environmental factors on the retention rate of PFS were explored using the Geo-Detector model. Table 5 shows the results of the q-values of the interaction influence between the ecological factors. The results show that there are only two types of interaction influence: the bivariate and nonlinear enhancement effects. There were 19 pairs of interaction factors with a nonlinear enhanced interaction effect in 2017, with FW and NMI being the only pair of interactive factors with a q-value > 0.90 (q = 0.92), indicating the interaction of FW and NMI had the greatest explanatory power of 92% for the spatial heterogeneity of PFS retention rate (*P* < 0.05). There were 10 pairs of interaction factors with a nonlinear enhanced interaction effect in 2018. Among these, PTI and FW was the only pair of factors with an interaction q-value > 0.90 (q = 0.96), indicating that the interaction of PTI and FW had the greatest explanatory power of 96% for the spatial heterogeneity of PFS retention rate (*P* < 0.05).

**Table 5:**
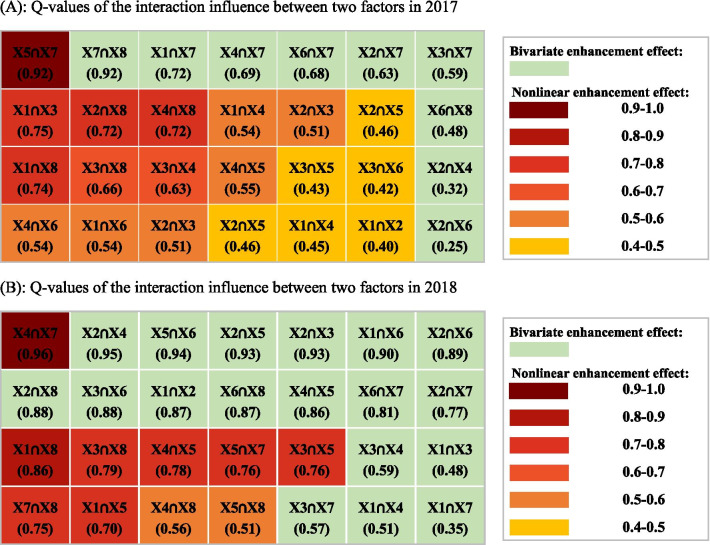
Q-values of the interaction influence between two factors after 12 months in 2017 (**A**) and 24 months in 2018 (**B**)

**Fig. 5:**
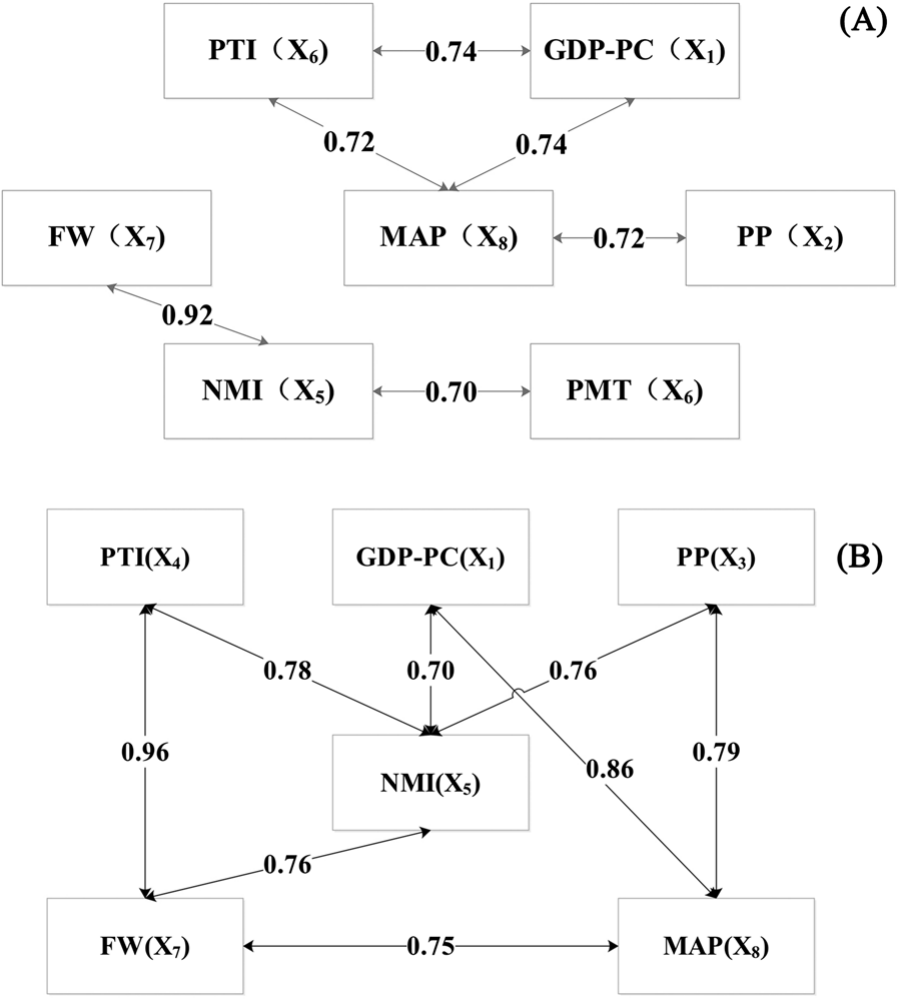
Network diagram of the interactive factors whose interactive q-values were > 0.70 after 12 months in 2017 (**a**) and 24 months in 2018 (**b**)

Figure 5 displays a network diagram of the interactive factors whose interactive q-values were > 0.70. The result indicate that MAP had the greatest number of other factors (RPP, PTI, and GDP-PC) with a nonlinear enhanced interaction > 70.0% after 12 months. Further, NMI had the greatest number of other factors (PP, PTI, GDP-PC, and FW) with a nonlinear enhanced interaction > 70.0% after 24 months.

Despite these results, our study had some limitations. As time progresses, the loss rate might also change. Accordingly, we will continue to monitor the loss rate of PFS in the future.

## Discussion

Since the 1980s, many studies [10, 12] have investigated the effectiveness of PFS, most of which involved controlled experiments under ideal conditions. The retention rate of PFS and the reduction rate of dental caries were often used as an evaluation index for preventing dental caries. The first permanent molar was randomly selected on one side for PFS, while the same arch on the other side was chosen as the control group without intervention. However, research under ideal conditions may not truly reflect the effect of PFS in the population under real conditions. Moreover, few studies have explored the influence of ecological factors in PFS.

The purposes of this study are to evaluate the retention rate and effectiveness of PFS under non-ideal conditions and to explore the impact of ecological factors. This study is based on the work conducted by NODIPC, where PFS were used by dentists on a large scale on children aged 7–9 years in hospitals and dental clinics. In the busy daily work of the dentist, they could seal dozens of children’s teeth with PFS.

### The retention of PFS and the prevalence rate of caries

The results indicated that the retention rate of PFS decreased over time. The full and partial retention rates were 81.6% and 13.4% after 12 months, respectively. In comparison, the full and partial retention rates were 75.1% and 14.2% after 24 months, respectively. Tu Rui et al. [22] demonstrated that the annual incidence of caries of the first permanent molars of children aged 7 years in China was 6.75%. Under realistic conditions, our study indicated that the incidence of caries and DMFT of the first permanent molars sealed by PFS were 2.06% and 0.07 after 12 months and 4.46% and 0.16 after 24 months, respectively. The results also demonstrate that the implementation of PFS can effectively reduce the risk of caries.

We also studied the relationship between the retention of PFS and gender and region through a chi-square analysis. The results indicate there was a significant difference in the retention rate according to gender after 24 months and the retention rate of boys was higher. This result may be due to the physical growth differences between boys and girls. Further, the physical development of girls occurs earlier than in boys and the eruption time of the first permanent molars in girls are earlier. Accordingly, the risk of sealant loss in girls is higher than in boys [23].

In 2017 and 2018, the retention rate of the occlusal surface was significantly higher than the retention rate of the buccal/palatal surface. The buccal groove of the first permanent molar has a simpler shape than occlusal surface, shallower in position, and not perpendicular to the occlusal surface, meaning it is easily contaminated by saliva during operation. Therefore, buccal/palatal sealant is not well retained and is easily lost. It reminds us that we should pay more attention to the groove of the buccal/palatal surface, isolate moisture, and standardize each step.

Anson concluded that the reasons for PFS loss were as follows: uncooperative children during the operation, not fully erupted teeth, difficulty in isolating moisture, and insufficient acid erosion. Therefore, standardized clinical operation is the key to the success of PFS [24]. In addition, if a child is uncooperative during the operation, the etched tooth surface can be easily contaminated; hence, it is necessary to communicate this with the child before the operation (or postpone treatment for extremely uncooperative children). Moreover, the moisture insulation measures such as suction, cotton rolls, or rubber barriers can improve the quality of PFS.

It is worth noting that the retention rate in rural areas was higher than in urban areas in Shanxi after 24 months, which is inconsistent with some studies [11, 25]. This phenomenon may be related to the type of organization implementing the project, such as if it is implemented in a public hospital or a private dental clinic. Among the 2201 teeth of urban children, 1507 teeth (68.5%) were sealed in private clinics and 694 teeth (31.5%) were sealed in public hospitals. Among the 1098 teeth of rural children, the proportions were 81.0% (889) and 19.0% (209), respectively. Compared with public hospitals, private dental clinics have relatively few channels to obtain customers. Accordingly, private dental clinics hope to obtain customer through this national project, meaning they are more motivated to implement the project and invest more labor and materials.

The results of the study also indicated there was no significant difference in the retention rate of PFS between the maxillary first permanent molars and the mandibular first permanent molars 12 and 24 months after PFS. This demonstrates that the retention rate is not related to a specific tooth arch, which is consistent with the results of other studies [26, 27].

### The spatial heterogeneity of the PFS retention rate

The spatial heterogeneity of the PFS retention rate was not clear after 12 months. Further, except for Datong City and Linfen City, the retention rates of other cities were > 90%. In comparison, the PFS retention rate after 24 months had greater stratified heterogeneity. Moreover, the PFS retention rate increased from north to south and the retention rates of Jincheng and Yuncheng in the southern region were both > 90%.

The study explored the interaction influence between the retention rate of PFS and eight ecological factors in the socioeconomic, medical resources, and natural environmental factors using the Geo-Detector model. The results indicate that natural environmental factors are important ecological factors that determine the spatial pattern after 12 months, while medical resources factors are important after 24 months. More specifically, FW plays a leading role in the spatial pattern of retention rate after 12 months and PMT after 24 months. We explored the interaction of ecological factors using the Geo-Detector model, where it was found that FW and NMI is the most influential interaction factor after 12 months while FW and PTI is the most influential after 24 months.

In the early 1980s, Shanxi Province of China was a major high-fluorine area with endemic fluorine poisoning. Although there was a significant decrease in FW after comprehensive prevention and control measures for reducing fluoride, some areas still have a higher FW than the national standard in Shanxi Province [28]. Further, studies [28, 29] have shown that tooth tissue will generate insoluble fluorapatite or calcium fluoride at finite concentrations of fluorine, which are deposited in the demineralized area of the tooth surface and promote remineralization. Hence, a small amount of fluorine has a preventive effect on caries. Research [30] indicates that the clinical effect of PFS combined with fluoridization is more effective than PFS alone. Moreover, fluoridization has the effect of preventing the loss of PFS. However, continuous intake of a large amount of fluoride will cause fluorine poisoning, which can result in dental and skeletal fluorosis. Well-known facts are that dental fluorosis affects enamel structure, producing several porosities on its surface affecting, pit and fissures of teeth [31]. This is important because patients with dental fluorosis are more likely to suffer losses after PFS. As shown previously, the uneven distribution of FW in Shanxi Province may cause the spatial heterogeneity of the PFS retention rate.

Shanxi Province is a vast territory with a large population, leading to uneven economic development among regions and unbalanced distribution of medical resources. Studies [32] have demonstrated that the distribution of medical technicians across Shanxi Province differs significantly. Further, medical resources are mainly concentrated in the southern and central regions of Shanxi, where the economy is developing rapidly and the population is denser. Accordingly, there is a particularly higher level of dental care in the south of Shanxi. For example, Yuncheng City government actively trained dental professionals as early as the 1980s to solve the problem of denture care in rural areas. Further, they have continued to develop rural oral health services. After more than 20 years of practice in dental prevention and treatment, a three-level dental prevention network with non-public ownership as the main body has been established in counties, townships, and villages, which has formed a dental prevention model for poor rural areas [21]. This imbalance of development has resulted in a limited number of medical institutions, a shortage of medical technicians, limited levels of medical technology, and a restricted approach to health services in the north. All of these factors ultimately cause a difference in people’s health across regions. This result suggests that Shanxi Province should strengthen medical and technician construction in the north, optimize the allocation of medical resources, and promote the balanced development of regional medicine.

Moreover, there are two main limitations of this study. The research period is not long enough and all samples in the study are from Shanxi Province. Although they are representative, they have obvious regional characteristics. More samples from other regions will be included in the future study.

## Conclusion

In Shanxi Province, the total retention rate of PFS conducted under NODIPC was 95.06% after 12 months and 89.30% after 24 months. We have demonstrated that children with PFS can effectively prevent dental caries in real life. Further, the project has made a great contribution to the cause of oral prevention. There was obvious spatial heterogeneity in the retention rate of PFS, which increased from north to south in 2018. The natural environmental factors are important ecological factors determining the spatial pattern after 12 months, which changed to medical resources factors after 24 months. It was determined that the NMI, PMT, and FW had a material effect on the spatial heterogeneity of PFS retention rate in Shanxi Province. Accordingly, the government should strengthen medical and technician constructions in the north, comprehensively control fluoride in water, optimize the allocation of medical resources, and promote the balanced development of regional medicine.

## Data Availability

Public access to the database is closed. However, the deidentified data set used and/or analyzed are available from the corresponding author on reasonable request.

## Abbreviations

PFS: Pit-and-fissure sealants
X1: GDP-PC (gross domestic product per capita)
X2: RPP (rural population percentage)
X3: PP (pupil percentage)
X4: PTI (the proportion of tertiary industry)
X5: NMI (number of medical institutions)
X6: PMT (percentage of medical technicians)
X7: FW (fluorine in water)
X8: MAP (mean annual precipitation)
NODIPC: The national oral disease intervention program for children
